# Use of Integra^®^ Dermal Regeneration Template Bilayer in Burn Reconstruction: Narrative Review, Expert Opinion, Tips and Tricks

**DOI:** 10.3390/ebj6030045

**Published:** 2025-08-18

**Authors:** Clemens Maria Schiestl, Naiem Moiemen, Patrick Duhamel, Isabel Jones, Marcello Zamparelli, Juan Carlos López-Gutiérrez, Simon Kuepper

**Affiliations:** 1 Univeristy Children’s Hospital Zurich, 8008 Zurich, Switzerland; 2 College of Medicine and Health, University of Birmingham, University Hospitals Birmingham Foundation Trust, Birmingham B15 2TT, UK; nmoiemen@me.com; 3 Percy Military Hospital, French Army Health Service, 92140 Clamart, France; patrick.duhamel6@orange.fr; 4 Val de Grâce School, 75005 Paris, France; 5 Department of Burns and Plastic Surgery, Chelsea and Westminster Hospital, London SW10 9NH, UK; isabel.jones2@nhs.net; 6 Plastic Surgery Unit and Pediatric Burn Centre, Santobono Pausilipon National Children’s Hospital, 80129 Napoli, Italy; m.zamparelli@santobonopausilipon.it; 7 Reconstructive Surgery and Burns Department, La Paz Children’s Hospital, 28029 Madrid, Spain; queminfantil.hulp@salud.madrid.org; 8 BG Klinikum Unfallkrankenhaus Berlin, Zentrum für Schwerbrandverletzte mit Plastischer Chirurgie, 12683 Berlin, Germany

**Keywords:** burn injury, dermal substitute, Integra^®^ Dermal Regeneration Template, skin regeneration, wound healing, reconstructive burn surgery, full-thickness burns, neodermis, skin grafting, burn wound management

## Abstract

Burn injuries remain a complex clinical challenge, particularly in reconstructive settings where donor sites are limited. Integra^®^ Dermal Regeneration Template (IDRT), a bilayer dermal substitute, facilitates neodermis formation and supports functional and aesthetic recovery following burn trauma. This narrative review and expert opinion synthesizes current literature and clinical experience on the application of IDRT in post-burn reconstruction. It discusses the biological mechanism of dermal regeneration, surgical protocols including wound bed preparation and grafting, and considerations for anatomical regions such as the face, torso, and limbs. The review emphasizes key factors influencing successful outcomes, including patient selection, timing, and multidisciplinary coordination. Potential complications, such as infection, hematoma, and poor graft adherence, are addressed along with prevention and management strategies. Special considerations for pediatric and elderly populations are also highlighted. Through evidence-based insights and illustrative case examples, this review aims to inform surgical decision-making and promote best practices in reconstructive burn care using IDRT.

## 1. Introduction

Burns are an important cause of morbidity and mortality worldwide, impacting millions of people each year. According to the World Health Organization (WHO), approximately 180,000 deaths annually are caused by burns [1]. Beyond the mortality impact, burns can lead to long-term physical disabilities, psychosocial trauma, and substantial economic burdens, affecting patients, their families, and healthcare systems. Adults with a history of burn injury have previously reported marked decrements in health-related quality of life compared to general population norms [2]. Several factors affect the quality of life for individuals who have experienced burns, including how they reintegrate into the community, the quality and appearance of their scars, and their self-perception [3]. Furthermore, worldwide, burn trauma in children is still one of the most common causes of lifelong disability due to function-limiting scars [4,5,6,7].

The gold standard for the initial treatment of severe burn injuries is to cover the wound with autologous epithelium as soon as possible after early excision to save the patient’s life. Additionally, the goal is to achieve a good functional and aesthetic outcome, enabling the patient to enjoy a better quality of life. Despite many advances in acute surgical treatment, there remains a significant need for reconstructive surgery after severe burn injuries to restore normal function and reduce stigmatization in the years following burn trauma.

Functional and aesthetic limitations due to extensive hypertrophic scars, contractures, and chronic wounds often require tailored clinical interventions [8] in the years following the initial treatment. Current treatment options for reconstruction after burn injury include autologous skin grafting, local tissue transfer, tissue expanders, and advanced surgical methods such as microsurgery. Among these, dermal regeneration templates have emerged as a notable option, offering the potential for improved patient outcomes [9,10].

Since 1981, when Burke and Yannas first published preliminary clinical results on the use of a bilayered ‘artificial skin’ as a dermal replacement in acute burns [11], Integra^®^ Dermal Regeneration Template Bilayer (IDRT) has been widely used and has contributed to the evolution of acute burn surgery as well as reconstruction after burns [9]. The efficacy and safety of IDRT in acute burn surgery are supported by a bulk of evidence [3]. Additionally, the data on IDRT applications in reconstructive surgery after burns show promise [3,9] and are the focus of this publication.

This narrative review aims to provide practical guidance to fellow plastic surgeons by explaining indications and procedures, ultimately seeking to enhance patient outcomes through informed and evidence-based strategies using IDRT in reconstructive surgery after burns. Although this review primarily addresses plastic surgery perspectives, it is important to acknowledge that in several countries burn surgery is also performed by general surgeons and hand surgeons (orthopedic surgeons), whose expertise plays a critical role in multidisciplinary burn care. By integrating literature-based clinical evidence with expert opinions and shared clinical experiences, we aim to advance the field of reconstructive surgery.

## 2. Indications

As the first dermal replacement with over 25 years of application in reconstructive surgery, IDRT has been recommended for a variety of clinical scenarios where conventional grafting may be limited or where enhanced outcomes are desired [12]. The use of a dermal template in combination with an autologous split-thickness skin graft (STSG) results in a significantly thicker dermal layer in the reconstructed area, closely resembling the natural dermis [12]. In contrast, the thin dermal layer formed after STSG alone often resembles scar tissue rather than the normal dermis.

The role of the dermal component in reconstruction is vital, influencing both functional and aesthetic outcomes. Clinical evidence and expert opinions may consistently support the effectiveness of IDRT in various reconstructive procedures, making it a valuable option when traditional grafting may be insufficient.

In the initial surgical treatment of full-thickness burns, IDRT is a recognized adjunct to autologous STSGs, supported by randomized controlled trials and nonrandomized studies [13,14,15]. A multicenter randomized trial in major burns reported a median take rate of 80% for IDRT in 106 patients, including both children and adults [13].

However, this article focuses on the use of IDRT in reconstructive surgery after burns in both adults and children. In burn reconstruction, IDRT has been shown to improve both functional and aesthetic outcomes, reduce hypertrophic scarring, and enhance skin appearance [14]. Additionally, IDRT helps restore normal skin structure and minimize contractures and scar-related impairments [14].

IDRT provides a protective matrix supporting tissue regeneration and coverage after burns involving exposed bone, tendon, or joint [16]. It facilitates dermal regeneration, promotes wound healing, and may reduce scarring as well as the need for microvascular flap reconstruction. For chronic burn wounds and non-healing ulcers, which are also at risk for malignant transformation (Marjolin ulcers), IDRT offers an alternative approach by promoting the formation of a vascularized neodermis, thereby supporting improved healing [16,17].

Notably, the functional improvement observed after burn scar excision and dermal restoration in pediatric reconstruction is significant. The expanding body surface area in children presents a distinct challenge in post-burn reconstructive surgery.

Given that IDRT is a multi-stage and time-consuming procedure, proper patient selection, tailored surgical techniques, and meticulous postoperative care are essential to maximize its benefits in burn reconstruction [9]. In addition, the relatively high cost of IDRT may restrict its use in certain healthcare settings, particularly where cost-effectiveness is a major concern.

## 3. IDRT for Different Burn Areas and Locations

The use of IDRT extends across multiple surgical disciplines, including plastic surgery, general surgery, and orthopedic hand surgery, depending on local healthcare structures and training systems. The application of IDRT is versatile and can be adapted to various anatomical locations, each with specific considerations based on literature-based clinical evidence and expert opinions. There are published studies using IDRT for burns reconstruction surgery in many areas of the body, including the face and neck, torso, and limbs. Here, we present the current evidence and some clinical insights for application at these sites [3].

### 3.1. Face and Neck

Reconstruction of the face and neck requires aesthetic precision and minimal scarring. IDRT has proven effective in enhancing aesthetic outcomes by promoting uniform dermal regeneration, making it suitable for visible areas where scar appearance is a concern.

For neck reconstruction, two studies reported improved outcomes—one a case report [18], and the other a cohort of 20 patients, four of whom had neck sites treated [19].

In 2005, Klein et al. published the first study on facial reconstruction using IDRT, involving 12 patients with deep facial burns. The authors found IDRT to be a viable option for patients with extensive burns and limited donor sites [20]. A more recent study of a 45-year-old woman with a facial burn from a clothes iron demonstrated the successful use of single-layer IDRT. The graft integrated smoothly without complications, infections, or the need for revision surgery. Despite the need for careful technique and preparation, this approach shortened operating time and improved clinical outcomes [21].

Histological studies further highlight IDRT’s regenerative capacity in facial and neck reconstruction. Moiemen et al. analyzed 20 patients with 30 reconstructive sites, demonstrating four phases of dermal regeneration—imbibition, fibroblast migration, neovascularization, and remodeling. Full vascularization occurred within four weeks. Although adnexa, nerve endings, and elastic fibers were absent, newly formed collagen resembled normal dermis. Patients reported enhanced motion and improved scar appearance [22].

**Figure 1:**
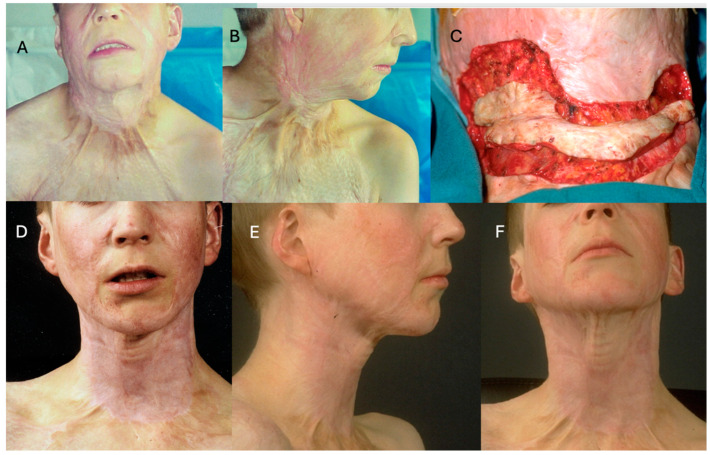
(**A**,**B**): Preoperative evaluation of a 14-year-old boy with a burn injury sustained 2 years earlier, presenting with a prominent facial scar and a sternomental contracture. (**C**): Intraoperative image showing defect size following scar excision. The excised scar is on the wound to demonstrate the size of the defect in relation to the excised scar. (**D**): 6-month postoperative follow-up demonstrating improved contour and scar maturation. (**E**,**F**): 12-month follow-up illustrating further enhancement in texture and scar appearance.

These findings indicate that IDRT is a valuable option for complex reconstructions of the face and neck, where both function and aesthetics are essential. A case from the author’s clinical experience shows the treatment of a 14-year-old boy with a prominent facial scar and a sternomental contracture, demonstrating progressive improvement at 6 and 12 months (Figure 1).

### 3.2. Torso

IDRT is a valuable option for reconstructing large burn areas on the chest, abdomen, and back. It provides a stable wound environment, promotes recovery, and improves skin elasticity, enhancing trunk mobility and functional outcomes.

Moiemen et al. reported reconstructive surgery in 20 patients, including procedures at two chest and two abdominal sites. Although site-specific data were not detailed, the cohort showed a 72% improvement in movement, a 62% increase in skin softness, and a 59% enhancement in appearance postoperatively [23].

One of the earliest applications of IDRT for chest reconstruction involved breast resurfacing after childhood burns. In this study, 92% of 12 patients with anterior chest burns reported high satisfaction with postoperative results [22].

**Figure 2:**
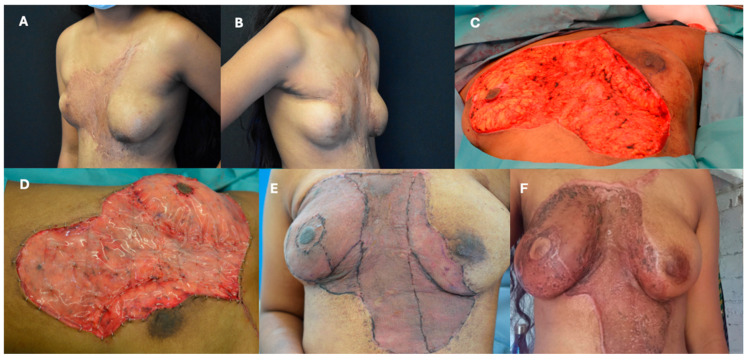
(**A**,**B**): Preoperative evaluation of a 16-year-old adolescent with a deforming scar from a scald injury sustained at age 2 years. (**C**): Intraoperative image after scar excision. (**D**): Defect coverage with IDRT during surgery. (**E**): Postoperative view 4 days after silicone layer removal and neodermis coverage with a thin split-thickness skin graft. (**F**): 12-month follow-up showing significant improvement in contour and scar maturation.

A case from the author’s experience highlights the use of IDRT in torso reconstruction. Figure 2 shows a 16-year-old adolescent with a severe post-burn scar from a childhood scald injury. Significant improvement was observed at 4 days and 12 months postoperatively.

### 3.3. Limbs

The limbs are particularly prone to functional impairment following burn injuries, with the axilla, hands, and lower extremities vulnerable to contractures and scarring that can severely limit mobility. Reconstructive approaches must address both function and aesthetics to optimize patient outcomes.

Axillary burns present a significant challenge due to contractures that restrict shoulder abduction and affect aesthetics [24]. Early intervention with splinting and exercises can help prevent contractures, but surgical release is often required once they develop. Achieving full functional release of the shoulder is critical, though post-surgical coverage can be complex and carries a high risk of complications.

IDRT has demonstrated positive functional and aesthetic outcomes in axillary reconstruction. In a study of four patients, no perioperative complications were reported, and shoulder mobility improved significantly postoperatively [25]. A larger study involving 20 patients (seven with axillary reconstructions) also showed improved skin quality and appearance [18]. Comparative studies of local flaps, IDRT, and free tissue transfer for axillary, elbow, and wrist contracture release have shown that all techniques when applied correctly, yield favorable long-term results [26]. Additional case reports further highlight improved outcomes following axillary IDRT application [18,27].

Hand burns can lead to severe disability and impact psychological, social, and professional well-being. Retractile scarring from insufficient coverage can limit movement [28]. IDRT has been extensively used in hand reconstruction, consistently showing favorable results. Dantzer et al. reported a 100% success rate for IDRT-treated hand burns, with supple tendon engraftment and preserved joint articulation, allowing for functional recovery [15,29]. A separate study involving 29 hands treated with IDRT showed high engraftment rates, minimal complications, and skin characteristics resembling normal dermis [30].

A retrospective review of 17 burned hands (14 patients) demonstrated full range of motion in 15 cases, flexible skin in 88%, and a 92.8% functional evaluation score according to the 400-point Hand Assessment (HA). No grip strength differences were found between treated and unaffected hands, with 79% of patients returning to work [31]. In traumatic hand injuries with exposed bone, joints, or tendons, IDRT grafting around 26 days post-application resulted in functional (total range of motion, TAM) and aesthetic success in 13 of 15 cases [32].

**Figure 3:**
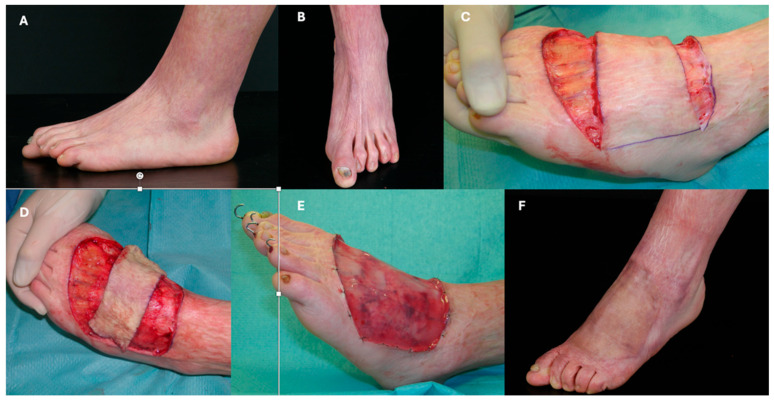
(**A**,**B**): Preoperative evaluation of an 18-year-old female patient with a prominent scar on the dorsum of the foot following a burn injury sustained at age 3 years. (**C**): Intraoperative image after scar incision. (**D**): Resulting defect size after full scar release. (**E**): Defect coverage with IDRT; K-wires were inserted to maintain toe extension. (**F**): 12-month follow-up demonstrating significant functional and aesthetic improvement.

Lower limb burns pose unique reconstructive challenges, especially in the feet and ankles, where contractures can impede walking. IDRT has been effectively used to restore mobility and improve aesthetics. A case from the author’s clinical experience illustrates IDRT application for a severe post-burn scar on the dorsum of the foot in an 18-year-old female. The defect was released and covered with IDRT, with notable improvement at 12 months (Figure 3).

These findings highlight IDRT’s versatility in limb reconstruction, demonstrating its ability to restore function and improve appearance in complex anatomical areas prone to contractures and mobility limitations.

## 4. Protocols

### 4.1. Protocols and Their Changes over the Last 25 Years

Since the approval of IDRT, protocols have evolved significantly, shifting from acute burn treatment to broader applications in reconstructive surgery [33]. Early protocols focused on frequent dressing changes, but current practices, shaped by clinical experience, recommend dressing changes every 4–7 days. This evolution reflects an improved understanding of dermal regeneration and postoperative care. A summary of the key aspects of the IDRT application is provided in Table 1.

**Figure 4:**
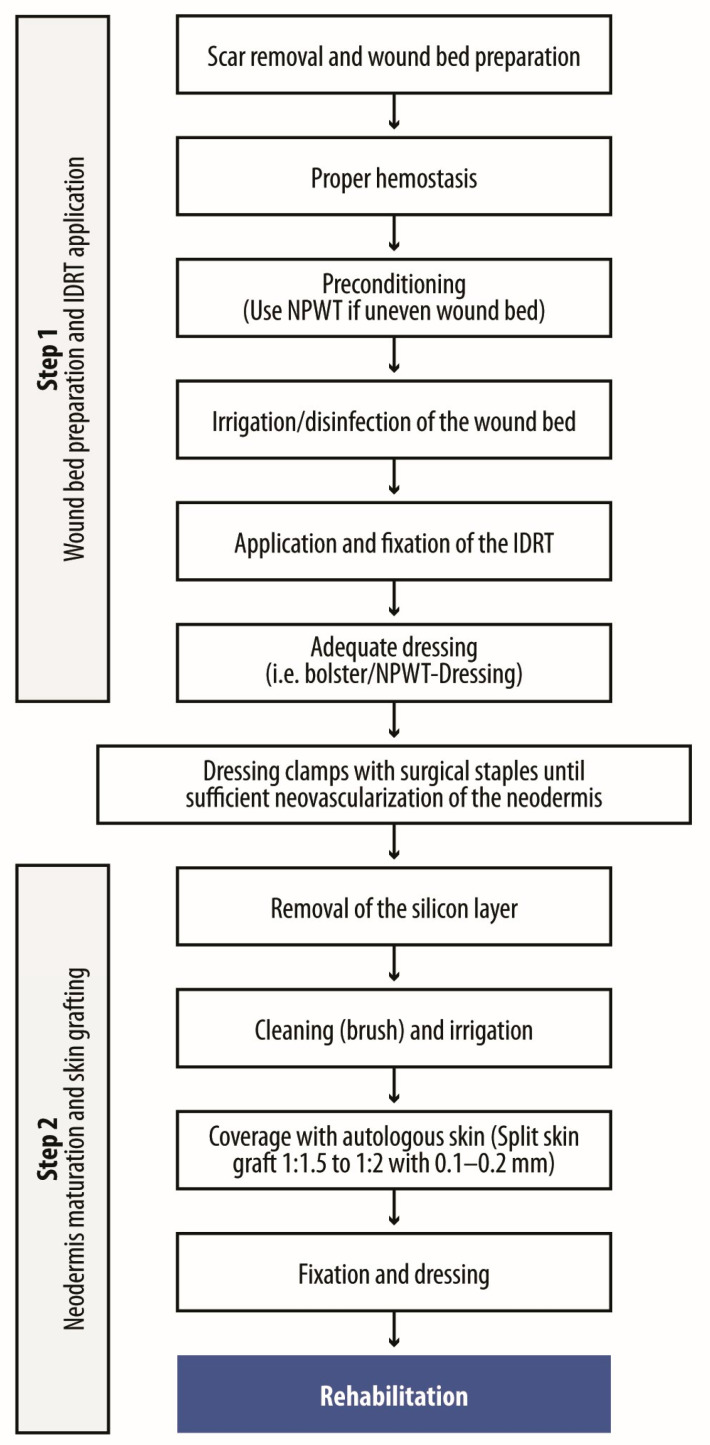
General protocol for post-burn reconstruction using IDRT.

The two-step surgical process involves wound bed preparation and IDRT application, followed by neodermis maturation and grafting (Figure 4). Initially, scar resection was limited to the affected area, but broader resections are now favored, particularly around joints, to reduce contractures and enhance movement. This approach, often referred to as creating a ‘recruitment zone’, improves flexibility and functional outcomes.

### 4.2. Wound Preparation and IDRT Application

Scar excision is performed using a scalpel or electrocautary. Hemostasis is essential to prevent hematomas beneath and under the template, with fibrin glue recommended to minimize bleeding [34]. In cases of irregular wound beds or poor vascularity, negative pressure wound therapy (NPWT) may be applied for several days to level the wound bed or enhance vascularization before IDRT placement [35]. Following thorough disinfection, the IDRT is secured with staples parallel to the edges and centrally as mattress sutures. Vacuum-assisted closure (VAC), increasingly used since 2001, improves fixation and reduces complications, particularly in pediatric cases [36,37].

### 4.3. Neodermis Maturation and Grafting

**Figure 5:**
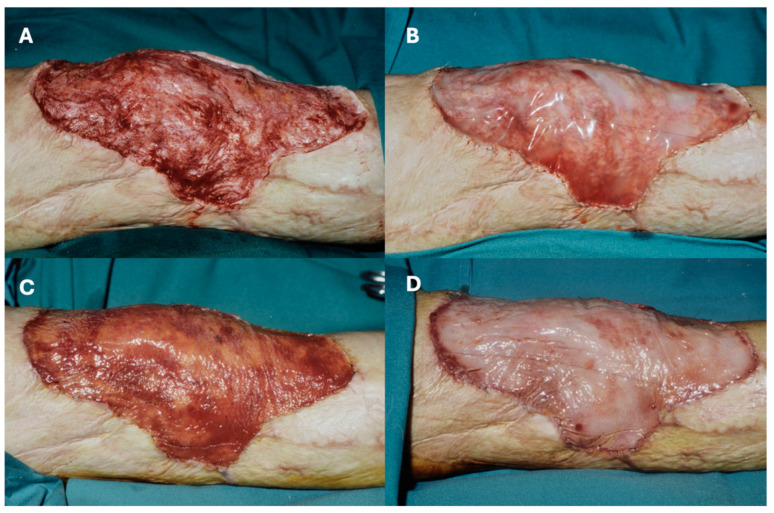
(**A**): Intraoperative image after excision of a scar above the knee. All scar tissue has been removed; (**B**). Intraoperative image shows IDRT directly after application. (**C**). Intraoperative image shows IDRT 3 weeks after application. A ‘neodermis’ can be seen through the silicone film, mainly appearing red. (**D**). Intraoperative image shows a clear color change from reddish to peachy after 5 weeks, a clear sign of sufficient vascularization of the neodermis.

Vascularization of the neodermis is slower in reconstructive settings, typically requiring 4–6 weeks (compared to 21 days in acute burns). A shift in color from red to peach signals adequate vascularization, at which point the silicone layer is removed, and a thin STSG is applied. Mesh grafts (1:1.5 or 1:2) are effective, but sheet grafts are preferred for optimal aesthetics. Figure 5 illustrates this process, demonstrating vascularization and the color shift at five weeks post-application.

### 4.4. Postoperative Care

Following STSG application, physiotherapy, splinting, and pressure garments are introduced after approximately two weeks to promote mobility and tissue maturation.

### 4.5. Special Considerations and Challenging Anatomical Areas

Customized protocols are essential for complex regions, such as the face, neck, hands and axilla. In these areas, IDRT must conform precisely to anatomical contours. For joints, slight flexion is maintained during application to facilitate movement. In the axilla, arm abduction and flexible dressings are key to preventing contractures.

## 5. Dressings

The success of IDRT in burn reconstruction heavily depends on proper dressing techniques, which create an optimal healing environment, protect against infection, and maintain moisture balance [38]. Dressings play a critical role in supporting dermal regeneration, preventing contamination, and ensuring patient comfort and mobility [39]. A summary of key dressing types and their roles is provided in Table 2.

### 5.1. Types of Dressings and Their Roles

Several types of dressings are used alongside IDRT, each with distinct advantages. Silicone-based dressings minimize pain and trauma during changes but require frequent monitoring in high-mobility areas. Foam dressings absorb exudate and maintain moisture but may need frequent replacement if bulky. Hydrocolloid dressings reduce dressing change frequency but can adhere too tightly to the wound bed, risking disruption during removal [40,41]. Antimicrobial dressings lower infection risk in high-contamination burns, though prolonged use may delay healing due to cytotoxicity [42]. Non-adherent dressings protect the wound but may lack sufficient absorbency, necessitating additional layers [41]. Besides traditional and advanced dressings, it is important to mention the use of NPWT as an adjunct therapy. If well calibrated in terms of duration and intensity, the use of NPWT helps ensure intimate contact between IDRT and the wound bed and can drain away excessive fluids from the wound.

### 5.2. Dressing Changes and Monitoring

Dressing changes should be performed every 3–7 days, depending on wound condition and exudate levels. More frequent changes may be required if excessive exudate or infection is present. Applying the initial dressing under sterile conditions immediately post-IDRT can help reduce contamination risk.

During changes, non-adherent or silicone-based dressings help minimize trauma to the wound. Regular wound inspection is essential for early detection of infection. Infected wounds may benefit from antimicrobial dressings or topical antibiotics according to approved indications. Maintaining appropriate moisture levels supports regeneration, while bulky dressings should be avoided to ensure patient compliance and comfort.

### 5.3. Post-Grafting Dressing Protocol

Following STSG application, dressings should be changed 5–7 days postoperatively. Care must be taken to avoid disturbing the delicate graft. Monitoring infection or graft failure is crucial. Absorptive dressings should be used to manage high exudate, and antimicrobials can be applied if infection is suspected.

## 6. Complication Management

Despite its clinical benefits, the use of IDRT is associated with a non-negligible risk of complications, particularly infections and partial graft loss. Published series report infection rates ranging from 10–20% and occasional hematoma or seroma formation [14,28,29]. These events may compromise integration and often require prompt management, ranging from antimicrobial therapy to partial template replacement. A summary of key prevention and management strategies is provided in Table 3.

### 6.1. Infections

Infections can undermine IDRT integration and healing. Preventive measures include strict aseptic technique during application and prophylactic antibiotics for high-risk patients. Regular wound inspection is essential. Suspected infections should be confirmed through microbiological tests before treatment. Local wound care, topical antimicrobials, and systemic antibiotics are standard interventions. In severe cases, infected tissue may require debridement, but conservative approaches that preserve viable portions of the IDRT are preferred. Full removal of the template is reserved for critical infections or sepsis.

### 6.2. Hematomas

Hematomas beneath the IDRT may impair integration and increase infection risk. Preventative measures include meticulous hemostasis and the use of fibrin glue during surgery. Postoperative monitoring, especially in the first 48 h, allows for early detection. Fluid hematomas can be aspirated with a needle and syringe, while solid hematomas require gentle incision and clot removal. Persistent bleeding may necessitate epinephrine application or electrocautery before reattaching the IDRT.

### 6.3. Lack of Adherence

Poor adherence can result from incomplete contact between the IDRT and the wound bed, excessive movement, or inadequate fixation. Prevention involves ensuring full contact during application, using NPWT, and securing the template with sutures or staples. If adherence fails, reapplication of the IDRT or NPWT can stabilize the wound and improve outcomes.

### 6.4. Poor STSG Take Rate

A poor take rate for the STSG applied over the IDRT can occur due to inadequate vascularization or infection. Ensuring complete vascularization before grafting and addressing inflammation or infection beforehand is essential. Managing moisture and restricting movement during the early postoperative phase helps graft adherence. In cases of graft failure, revision surgery or alternative grafting techniques may be necessary. Hyperbaric oxygen therapy can support vascularization and improve graft success.

### 6.5. Shrinkage

Shrinkage of regenerated tissue can affect function and appearance. Early mobilization, physical therapy, and splinting are critical preventive measures. If shrinkage occurs, intensive therapy and stretching exercises can mitigate contractures. Severe cases may require surgical release or revision procedures to restore range of motion and tissue elasticity.

## 7. Tips and Tricks

### 7.1. Treatment Planning and Coordination

Reconstructive surgery using IDRT is complex, often reserved for cases where simpler techniques are inadequate. Success requires careful coordination among a multidisciplinary team, as well as consideration of the patient’s environment, including family and caregivers.

Meticulous planning is essential, particularly for challenging areas such as the face, neck, and near orifices. In such cases, assembling the full surgical team, caregivers, and family is advised. This collaborative approach is vital, as patient behavior during and after surgery—such as immobilization of mobile areas—can influence outcomes.

Organizing preoperative meetings involving all stakeholders may seem resource-intensive but can ultimately reduce complications and improve efficiency, justifying the associated costs [43].

### 7.2. Learning Curve

Clinicians must navigate a learning curve to achieve optimal results with IDRT. Hands-on workshops, mentorship, and training are essential to understand the nuances of application and postoperative care [44]. Expertise should be developed, starting with easier cases that involve simpler anatomical sites (e.g., upper trunk) and progressing to more complex regions. Surgeons should seek guidance from experienced colleagues, especially during initial cases.

### 7.3. Tailored Approach

Each patient presents unique challenges, necessitating a customized treatment plan, which is crucial for success and should be obligatory before going into surgery. A thorough assessment of patient health, prior burn severity, loss of function, and anatomical considerations is critical. Individualized protocols for fixation and postoperative care improve integration and healing.

Flexibility during surgery, with adjustments based on intraoperative findings, enhances success rates. Adapting the approach to the patient’s evolving needs ensures better long-term outcomes.

### 7.4. Wound Bed Preparation and Management

A clean, viable wound bed is essential for successful IDRT application [45]. Thorough debridement to remove necrotic tissue prevents infection and facilitates integration. Hemostasis is equally crucial to avoid hematoma formation, which can disrupt template adherence.

Hemostasis can be achieved through epinephrine, electrocautery, thrombin spray, or thrombin-soaked gauze [45]. The wound must also maintain appropriate moisture balance to support cellular ingrowth and vascularization. Effective dressing management prevents maceration and maintains a suitable healing environment [46].

By ensuring a well-prepared wound bed—whether fascia, fat, dermis, muscle or bone—IDRT adherence is optimized, improving functional and aesthetic outcomes.

## 8. Conclusions

IDRT has been used in reconstructive surgery for burn scar treatment for over 25 years. The use of a dermal template to prepare the wound bed and support neodermis formation represents a valuable advancement in reconstructive surgery. IDRT has shown advantages in reconstructing extensive defects following severe burn injuries, particularly in cases where other methods may be limited.

Surgeons benefit from familiarity with a range of reconstructive techniques, including those with established safety and efficacy profiles, such as IDRT. This review aims to contribute to the education and training of surgeons who may be less experienced with this technology. Although the recommendations provided are based on current evidence, ongoing research and broader clinical consensus remain necessary to fully assess and refine the role of IDRT in burn reconstruction. At the same time, an important consideration is the high cost of IDRT, which remains a limiting factor in many countries and underscores the need to balance clinical benefits with economic feasibility when planning reconstructive strategies.

## Figures and Tables

**Table 1:** Summary of IDRT application protocols for detailed procedural steps.

Protocol Stage	Key Steps and Considerations	Notes
Preoperative preparation	Comprehensive patient assessment (comorbidities, burn severity, anatomical site)	Infection control essential
	Treat existing infections to minimize postoperative complications	
	Plan excision area to include surrounding flexible tissue for improved mobility	
	Consider NPWT preconditioning to improve vascularization, especially for irregular wound beds	
Wound Bed preparation	Scar excision (extensive resection improves outcomes in joint areas)	Electrocautery or scalpel-based
	Ensure meticulous hemostasis to prevent hematoma formation	Fibrin glue recommended
	Disinfect wound thoroughly	
IDRT application and fixation	IDRT cut to fit wound area	Immediate application post-excision
	Secure with staples (parallel to edges, centrally placed mattress sutures)	
	Consider controlled vacuum-assisted closure (VAC) for fixation, especially in pediatric cases	Improves adherence, reduces movement and the likelihood of seroma due to reduced movement or sliding
Neodermis maturation	Monitor vascularization (color shift from red to peach indicates readiness for next stage)	Typically 4–6 weeks for maturation
	Delay silicone removal until full vascularization is evident	Longer than acute burn protocols
Silicone layer removal and grafting	Gently debride poorly vascularized areas with a brush	Sheet or mesh graft 0.1–0.2 mm thick
	Apply thin STSG to neodermis	Mesh expansion 1:1.5 or 1:2
	Sheet graft preferred for aesthetic regions	
Postoperative care	Change dressings every 4–7 days (not daily)	Reduces infection risk
	Monitor for infection, fluid buildup, and displacement	Hydration and nutrition critical
	Introduce physiotherapy and splinting to maintain mobility post-STSG	Begin after 2 weeks
	Use pressure garments until full tissue maturation	
Special considerations	Pediatrics: Smaller IDRT, gentle fixation, parental involvement	
	Elderly: Monitor for vascular disease, ensure soft fixation and optimize nutrition	
	Challenging Areas:	
	Face/neck: Custom-cut IDRT, sutures/tissue adhesives for contouring	
	Hands/fingers: Fine sutures, splints to maintain movement	
	Elbows/knees: Apply with joint in slight flexion, elastic bandages	
	Axilla: Maintain arm abduction, flexible dressings	

**Table 2:** Summary of Dressing Techniques and Management for IDRT.

Aspect	Key Points & Considerations
Goals of dressing management	Support dermal regeneration; prevent contamination and infection; protect wound from trauma; ensure comfort, mobility, and moisture balance.
Silicone-based dressings	Non-adherent, minimize pain/trauma during changes; require frequent monitoring in high-mobility areas.
Foam dressings	Absorb exudate, maintain moist environment; can become bulky, may require frequent changes.
Hydrocolloid dressings	Absorptive, reduce dressing frequency; may adhere too strongly, risking disruption during removal.
Antimicrobial dressings	Reduce infection risk (high-contamination sites); prolonged use may delay healing (cytotoxicity).
Non-adherent dressings	Protect wound bed, minimize trauma; may not absorb enough for highly exudative wounds.
Negative pressure wound therapy	Adjunct to improve adherence and fluid control; duration and intensity must be carefully calibrated.
Dressing change frequency	Every 3–7 days, adjusted for exudate or infection.
Initial post-STSG change	At 5–7 days post-application; avoid trauma to graft during early changes.
Infection monitoring & management	Inspect regularly; use antimicrobial dressings or topical antibiotics as needed.
Moisture balance	Essential for healing; foam or hydrocolloid dressings recommended.
Patient comfort	Ensure proper fit and compliance.

**Table 3:** Complication Management for IDRT.

Complication	Prevention	Management
Infection	Apply IDRT under sterile conditions Use prophylactic antibiotics for high-risk patients Ensure regular wound inspection	Confirm infection with microbiological tests before treatment Clean wound thoroughly and apply topical antimicrobials or systemic antibiotics Debride infected tissue conservatively; preserve IDRT when possible Reapply IDRT if wound bed improves post-infection
Hematoma	Achieve meticulous hemostasis during surgery Use fibrin glue or topical hemostatic agents Monitor closely in the first 48 h post-op	Aspirate fluid hematomas with a needle or cotton tip applicator For solid hematomas, incise and remove clots with gentle rolling motions Address persistent bleeding with epinephrine or electrocautery Re-secure IDRT after hematoma evacuation
Lack of adherence	Ensure full contact between IDRT and wound bed Use NPWT post-application to stabilize IDRT Avoid excessive movement at wound site	Reevaluate fixation (sutures, staples, or tissue adhesives) if adherence fails Consider reapplying IDRT with NPWT to promote stability
Poor STSG take rate	Confirm full vascularization of IDRT before STSG application Address infection/inflammation prior to grafting	Maintain moist environment and minimize movement post-graft Consider revision surgery or alternative grafting if failure occurs Use HBOT to enhance vascularization if needed
Shrinkage (tissue contraction)	Early mobilization and physical therapy Use splints or orthotic devices	Intensive physical therapy and stretching exercises Surgical release or revision if severe contractures develop
